# A low cartilage formation and repair endotype predicts radiographic progression of symptomatic knee osteoarthritis

**DOI:** 10.1186/s10195-021-00572-0

**Published:** 2021-03-09

**Authors:** Yunyun Luo, Jonathan Samuels, Svetlana Krasnokutsky, Inger Byrjalsen, Virginia B. Kraus, Yi He, Morten A. Karsdal, Steven B. Abramson, Mukundan Attur, Anne C. Bay-Jensen

**Affiliations:** 1 Rheumatology, Biomarkers and Research, Nordic Bioscience, Herlev Hovedgade 207, 2730 Herlev, Denmark; 2 Faculty of Health and Medical Sciences, University of Copenhagen, Copenhagen, Denmark; 3 Division of Rheumatology, NYU School of Medicine and NYU Langone Orthopaedic Hospital, New York, USA; 4 Clinical Development, Nordic Bioscience, Herlev, Denmark; 5 Division of Rheumatology, Department of Medicine, Duke University School of Medicine, Durham, USA; 6 Duke Molecular Physiology Institute, Duke University School of Medicine, Durham, USA

**Keywords:** Cartilage biomarker, Extracellular matrix, Joint space narrowing, Knee osteoarthritis, Matrix synthesis

## Abstract

**Background:**

Osteoarthritis (OA) is a disease with multiple endotypes. A hallmark of OA is loss of cartilage; however, it is evident that the rate of cartilage loss differs among patients, which may partly be attributed to differential capacity for cartilage repair. We hypothesize that a low cartilage repair endotype exists and that such endotypes are more likely to progress radiographically. The aim of this study is to examine the associations of level of cartilage formation with OA severity and radiographic OA progression. We used the blood-based marker PRO-C2, reflecting type II collagen formation, to assess levels of cartilage formation.

**Materials and methods:**

The type II collagen propeptide PRO-C2 was measured in the serum/plasma of knee OA subjects from New York University (NYU, *n* = 106) and a subcohort of the phase III oral salmon calcitonin (sCT) trial SMC021-2301 (SMC, *n* = 147). Risk of radiographic medial joint space narrowing (JSN) over 24 months was compared between quartiles (very low, low, moderate, and high) of PRO-C2. Associations were adjusted for age, gender, BMI, race, baseline pain levels, and baseline joint space width.

**Results:**

In both the NYU and SMC cohorts, subjects with low PRO-C2 levels had greater JSN compared with subjects with high PRO-C2. Mean difference in JSN between subjects with very low and high levels of PRO-C2 was 0.65 mm (*p* = 0.002), corresponding to a 3.4 (1.4–8.6)-fold higher risk of progression. There was no significant effect of sCT treatment, compared with placebo, on JSN over 2 years before stratification based on baseline PRO-C2. However, there were proportionately fewer progressors in the sCT arm of the very low/low PRO-C2 group compared with the moderate/high group (Chi squared = 6.5, *p* = 0.011).

**Conclusion:**

Serum/plasma level of type II collagen formation, PRO-C2, may be an objective indicator of a low cartilage repair endotype, displaying radiographic progression and superior response to a proanabolic drug.

**Level of evidence:**

Level III post hoc exploratory analysis of one longitudinal cohort and a sub-study from one phase III clinical trial.

## Introduction

Osteoarthritis (OA), the most common arthritis, is characterized by progressive cartilage destruction, deterioration of subchondral bone, and synovial inflammation [1, 2]. It affects 10–20% of the adult population and leads to debilitating pain, functional impairment, and disability among elders [3]. OA is a highly heterogeneous disease characterized by the involvement of single or multiple joints, variable clinical features, and biochemical/genetic characteristics [4], which suggests that multiple phenotypes and endotypes exist.

A phenotype is defined as the observable properties of an organism produced by the interactions of the genotype and environment. Patients with common characteristics are grouped together to guide therapy and management [5]. In contrast to a phenotype of a disease that is without any implication of a mechanism, an endotype identifies a specific biological pathway or a distinct pathophysiological mechanism explaining the observable characteristic of a phenotype. Endotypes are defined by specific cells or molecules in the blood, urine, and/or other biological specimens [6]. Although endotypic classification is a more specific and accurate way of defining patient subgroups, this method of classification is not yet uniformly used in the OA field [7, 8]. One simplistic and general example of a phenotype is knee pain, while the related endotype could be the synovial fluid concentration of a synovial pain marker. Determination of a disease endotype is possible when the observed changes during disease progression and/or patient response to treatment can be quantified by biomarkers [9]. Therefore, knee OA disease heterogeneity could be further explored with new biomarkers indicative of a particular endotype with distinct mechanistic pathways (e.g., low cartilage formation) and/or variable clinical presentations (e.g., radiographic fast progressors). Identification of such endotypes by validated biomarkers could assist in enabling a precision medicine approach for OA and eventually facilitate the development of targeted therapies for OA [7].

Recent data from the large-scale UK biobank identified genetic polymorphisms in eight genes associated with OA [10]. Three of these genes were linked to a cartilage formation/repair endotype, namely growth differentiation factor 5 (*GDF5*), fibroblast growth factor 18 (*FGF18*), and transforming growth factor-beta 1 (*TGF*-*β1*) [10], suggesting that cartilage formation, when impaired, may be associated with a higher level of OA structural disease progression due to repair attenuation. Thus, cartilage formation by chondrocytes may represent a convergent mechanism with cartilage repair pathways. By analogy to the liver fibrosis field, wherein a high fibrosis formation phenotype is more likely to respond to an antifibrotic therapy [11], we hypothesized that in OA, a lower cartilage formation endotype is more likely to respond to some OA treatments.

An N-propeptidase generated biomarker of type II collagen formation in serum/plasma, the N-terminal propeptide of collagen type IIB (PIIBNP, hereafter called PRO-C2) [12], can serve as a surrogate biomarker for cartilage formation [13], making it possible to investigate a potential cartilage repair endotype in OA. We recently demonstrated that PRO-C2 concentrations were lower in OA patients compared to healthy controls and could be induced by potential cartilage anabolic therapy [14].

In the current post hoc exploratory analysis of two independent cohorts of knee OA patients, we examined whether there was an association between baseline PRO-C2 blood levels and radiographic OA severity and progression.

## Methods

### Study design and participants

#### The NYU cohort

**Fig. 1:**
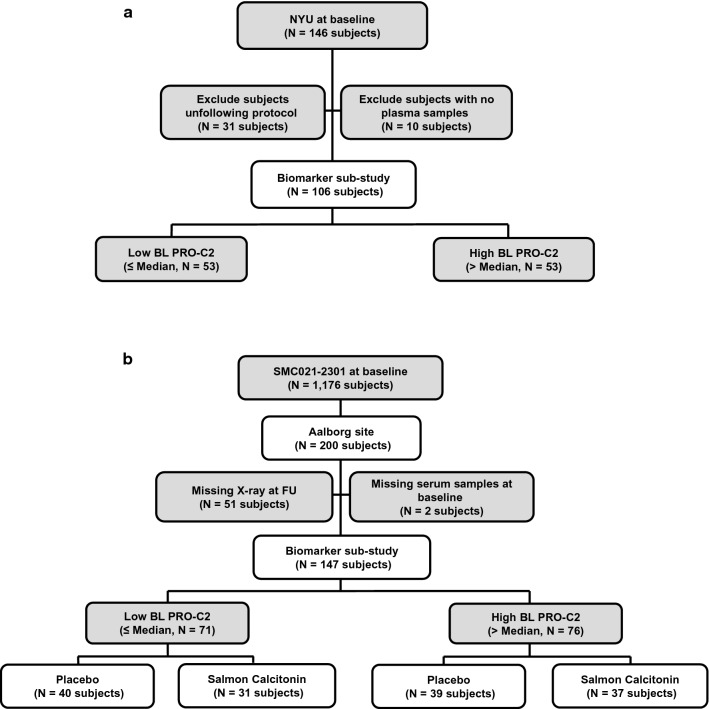
Flow diagrams for subjects included in the PRO-C2 post hoc analyses. **a** The NYU cohort. **b** The SMC cohort

The New York University (NYU) cohort with varying degrees of knee OA (*n* = 106) was recruited at New York University [15–18] (Fig. 1a). All patients underwent bilateral standardized weight-bearing fixed flexion posteroanterior knee radiographs using the SynaFlexer™ positioning frame (Synarc). Radiographic readings were done separately by two musculoskeletal radiologists blinded to patient information. Disagreements between the two readers were resolved by consensus. Radiographic progression was assessed by medial joint space narrowing (mJSN), based on the change in joint space width (JSW) of the signal knee at baseline and at 24 months. Medial joint space widths (mJSW) were measured at the mid-portion of the joint space via electronic calipers [15]. At baseline, all patients had complained of pain in the signal knee and met American College of Rheumatology (ACR) clinical criteria for knee OA. Non-fasting blood samples were collected for heparin-treated plasma and stored at −80 °C until biomarker measurement.

#### The SMC cohort

From a total of 1176 individuals in the phase III OA trial SMC021-2301 (clinicaltrial.gov: NCT00486434) testing the efficacy of oral salmon calcitonin (sCT), 200 participants recruited in one study center (Aalborg, Denmark) were selected for preliminary proof-of-concept [19, 20]. A total of 51 participants were lost at follow-up because they were missing 2-year x-rays and, 2 subjects had missing serum samples at baseline. This SMC subcohort, therefore, comprised 147 participants with knee OA (Fig. 1b). Both knees were examined during the study, but a signal knee was chosen prior to randomization based on the following inclusion criteria: KLG 2 or 3 of the medial tibiofemoral joint; a radiographic JSW of the signal knee ≥ 2.0 mm of the medial tibiofemoral compartment as measured on a radiograph; classification by ACR criteria as functional class I, II, or III; a Western Ontario and McMaster Universities Osteoarthritis Index (WOMAC) version VA3.1 [21] pain subscale (five questions, a score from 0 to 500 mm) score of ≥ 150 mm, and/or a WOMAC function subscale (17 questions, a score from 0 to 1700 mm) score of ≥ 510 mm. Weight bearing knee radiographs were performed using a non-fluoroscopic, standardized, quality-controlled method as described in the previous study [19] with the SynaFlexer™ positioning frame, and a fixed external rotation of both feet to ensure a similar standardized positioning of all patients. At baseline, all patients met ACR clinical and radiographic criteria for knee OA. For a detailed description of the study population and inclusion/exclusion criteria, see [19, 20].

### Assessment of PRO-C2

Plasma PRO-C2 was assessed at baseline in NYU cohort using a high sensitivity (hs) PRO-C2 electro-chemiluminescence ImmunoAssay (ECLIA) (Nordic Bioscience, Herlev, Denmark). Serum PRO-C2 was assessed at baseline and follow-ups (1 month, 6 months, 12 months and 24 months) in SMC pilot study. It is a competitive assay based on a monoclonal antibody specific to the epitope (QDVRQPGPKG) derived from the N-terminal propeptide region of the type IIB procollagen α1-chain [14]. We have previously developed the PRO-C2 enzyme-linked immunosorbent assay (ELISA), which, had some sensitivity limitations. Therefore, we converted the assay to the ECLIA platform, resulting in a seven-fold increased sensitivity [14]. PRO-C2 was measured in non-fasting plasma in the NYU cohort (sera samples were not available) and the placebo (PLB) and sCT arms of the SMC subcohort (fasting serum). The effect of fasting and non-fasting was tested in ten donor samples (Additional file 1: Fig. S1). The measurement was in duplicate and blinded to the clinical data in the SMC cohort, whereas single determinations were made in the NYU cohort due to the limited volume of the samples. The intra-assay coefficient of variation (CV) of PRO-C2 was 7.4%, and the inter-assay CV was 13.4%. The lower limit of detection (LLOD) was 0.08 ng/mL, defined as the concentration corresponding to 3 standard deviations (SD) above the mean of 21 determinations of the zero calibrator.

### Statistical analysis

This is a post hoc and explorative analysis to test the association between radiographic progression and the cartilage formation marker PRO-C2.

Baseline demographics and characteristics are presented as mean and standard deviation (SD) or as frequency and percentage (%). Comparison of age, BMI, gender, and race between cohorts was done by either Mann–Whitney test or by Chi squared test; cohort baseline VAS pain, KLG, and mJSW were compared by multiple regression analysis adjusting for age, BMI, gender, and race.

PRO-C2 data were normalized using logarithmic transformation. Correlations with age, BMI, gender, race, with VAS pain and JSW at baseline was performed using multiple linear regression in each of the cohorts.

Each of the cohorts was dichotomized into low and high levels of PRO-C2 separated by the median using all baseline data (Additional file 2: data Fig. S5). The difference in 2-year joint space narrowing (JSN) in the signal knee between low and high were analyzed in each cohort by ANCOVA with age, BMI, gender, VAS pain, and baseline medial joint space width (signal knee) as covariates. The “dose–response” association between PRO-C2 levels and JSN was investigated by first separating the subjects into quartiles (Q1–Q4) of PRO-C2 in the individual studies and then pooling the patients from both cohorts by quartile for purposes of assessing the association of baseline PRO-C2 with JSN. The odds ratio for progression between the PRO-C2 Q4 (reference) and the remaining quartiles was assessed by logistic regression, where progression was defined as JSN > 0 (any progression). The analysis was adjusted for the covariates.

Proportional difference in responders and non-responders between placebo and sCT arms of the SMC study in groups of low and high (median cut-off) levels of baseline PRO-C2 was tested by Chi square. Non-responders were defined as those with JSN > 0.35 mm which was based on the 2-year average JSN in the Osteoarthritis Research Society International/Foundation for the National Institutes of Health (OARSI/FNIH) OA biomarkers consortium [22].

All statistical analyses were performed with MedCalc version 19.1.7 (MedCalc Software, Ostend, Belgium), and graphing was done with GraphPad Prism version 8.3 (GraphPad Software, CA, USA). The significance level was set at α = 0.05, but reported to 0.1

## Results

### Study populations and baseline characteristics

Among the 147 patients enrolled in the NYU study, 31 were lost at follow-up, and 10 were excluded due to lack of plasma samples. The remaining 106 individuals were dichotomized according to median level (1480 pg/mL) of baseline plasma PRO-C2 (Fig. 1a). The 147 subjects in the sSMC subcohort were dichotomized based on median level (1960 pg/mL) of baseline serum PRO-C2 (Fig. 1b).

Demographic, clinical, and radiographic details of the 106 NYU and the 147 SMC participants are presented in Table 1. Mean age and BMI were higher in the SMC cohort compared with the NYU cohort. There were more female participants in the NYU cohort, but not significantly. There were only Caucasians in the SMC cohort in contrast to the NYU cohort where 66% were Caucasian, 25% Black, and 9% other. Approximately 21% of the NYU participants used NSAIDs at baseline, whereas none of the SMC participants did.

**Table 1:** Cohort description

Variables	NYU cohort (*N* = 106)	SMC cohort (*N* = 147)	Difference between cohorts (*p* value)
Age, mean (SD) years	61.0 (10.2)	63.6 (6.6)	0.026
BMI, mean (SD) kg/m^2^	26.6 (3.6)	29.0 (4.5)	0.0007
Gender, no. female (%)	69 (65)	82 (56)	ns
Race, no. White/Black/other (%)	70 (66)/26 (25)/10 (9)	147 (100)/0/0	< 0.0001
Pain, mean (SE) VAS 0-100 mm	42.1 (2.7)	48.2 (2.1)	ns
mJSW on signal knee, mean (SE) mm	3.63 (0.10)	3.31 (0.09)	0.022
KLG, Frequency (%)			
0–1	29 (27)	0	0.030
2	20 (19)	129 (88)	
3–4	57 (54)	18 (12)	

BMI: body mass index; KLG: Kellgren-Lawrence grades; mJSW: medial joint space width; ns: not significant; SD: standard deviation; SE: standard error; VAS: visual analog scale

After adjusting for age, gender, race, and BMI, there was no difference in the average pain score of the signal knee between the two cohorts (Table 1). Mean JSW of the signal knee was significantly higher in the NYU cohort after adjusting for age, BMI, gender, and race. The NYU cohort included patients with KLG 0 to 4 in the signal knee, whereas the SMC cohort only included patients with KLG 2 and 3 (Table 1).

Serum/plasma PRO-C2 levels were correlated with age (*r* = 0.41, *p* = 0.0001) and race (*r* = 0.34, *p* = 0.0010) in the NYU cohort and with BMI (*r* = 0.24, *p* = 0.0013) and JSW (*r* = −0.54, *p* < 0.0001) in the SMC cohort on univariate testing (Table 2).

**Table 2:** Correlation (multiple linear regression) between PRO-C2 and baseline characteristics

	Age	BMI	Gender	Race	Pain VAS*	mJSW*
NYU	0.41 (0.0001)	0.19 (ns)	0.17 (ns)	0.34 (0.0010)	0.06 (ns)	0.20 (ns)
SMC	− 0.10 (ns)	0.24 (0.0013)	0.11 (ns)	–	− 0.09 (ns)	− 0.54 (< 0.0001)

Data are shown as partial *r* (*p* value), where non-significant (ns) is *p* > 0.1

BMI: Body mass index; mJSW: medial joint space width; ns: not significant, VAS: visual analogue scale

*Signal knee

### Association between baseline PRO-C2 and medial joint space narrowing

**Fig. 2:**
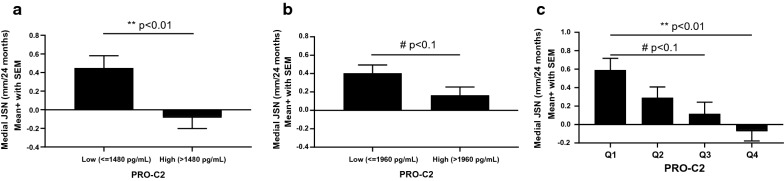
Association between baseline levels of PRO-C2 and 2-year radiographic progression (JSN). Patients were dichotomized based on the median to low and high levels of PRO-C2 and the degree of progression was compared in **a** the NYU and **b** the SMC cohort. **c** Assessment of “dose–response” of JSN as a function of PRO-C2 levels in the combined dataset (all subjects in NYU cohort and the placebo arm of SMC subcohort). Analyses were adjusted for age, BMI, gender, VAS pain, and baseline medial JSW using two-way analysis of covariates (ANCOVA). Data are presented as mean with standard error of mean (SEM). Asterisks: **p < 0.01; ^#^*p* < 0.1

Difference in two-year JSN was assessed in the NYU and SMC cohorts on the dichotomized data (Fig. 1). Patients with low PRO-C2 progressed significantly faster than patients with high PRO-C2 in the NYU cohort, mean difference was 0.52 mm (*p* = 0.0078) (Fig. 2a). Similarly, in the SMC cohort, JSN was greater in the low PRO-C2 group compared with the higher PRO-C2 group of the placebo arm: mean difference was 0.24 (*p* = 0.078) (Fig. 2b), however, this difference was of borderline significance. Scatter plots of the PRO-C2 distribution and summary statistics for each of the subgroups can be found in the Additional file 3: Fig. S2.

Next, we pooled the data of the two cohorts to assess the overall “dose–response” of JSN as a function of baseline PRO-C2 levels. The mean differences between the lowest level of PRO-C2 (Q1, very low) and the higher levels of PRO-C2 were: Q2 (low), 0.30 (ns); Q3 (moderate), 0.48 (*p* = 0.056); and Q4 (high), 0.65 (*p* = 0.0018) (Fig. 2c). These data indicate that radiographic progression is more pronounced in patients with low type II collagen formation, an effect that was independent of the covariates. Scatter plots of the PRO-C2 distribution can be found in the Additional file 3: Fig. S2.

### Subjects with very low levels of baseline PRO-C2 are more likely to progress

**Fig. 3:**
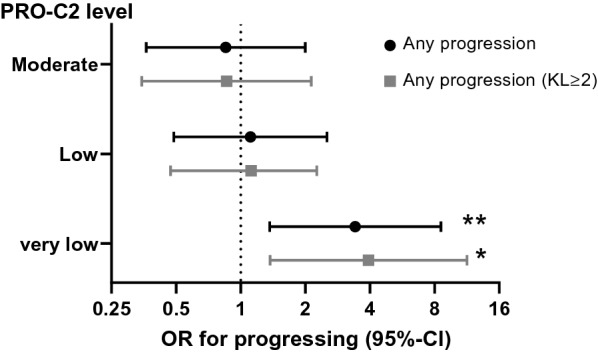
The odds ratio (OR) for progression in groups of patients with very low (Q1 from Fig. 2), low (Q2), and moderate (Q3) compared with high (Q4) levels of baseline PRO-C2. Progression was defined as any progression (JSN > 0) over 2-years in analyses including all subjects in NYU cohort and the placebo arm of SMC subcohort (*n* = 185, black) and individuals with baseline KL ≥ 2 (*n* = 156, grey). Analyses were adjusted for age, BMI, gender, VAS pain, and baseline medial JSW using logistic regression. Data are presented as OR and 95% confidence intervals (CI). Asterisks: **p < 0.01; *p < 0.05

We investigated the odds ratio (OR) for progression of the different groups using the high PRO-C2 group (Q4 in Fig. 2) as a reference. Participants in the very low group (Q1) were more likely to progress over the two-year period than the high group: OR 3.4 (1.4–8.6), *p* = 0.0087 (Fig. 3). A similar likelihood of progression was observed in the very low PRO-C2 group of participants with definite radiographic OA (KLG ≥ 2): OR 3.9 (1.4–11.3), *p* = 0.011 (Fig. 3). Subjects with low (Q2) and moderate (Q3) levels were not more likely to progress compared with those with high levels (Q4) (Fig. 3).

### PRO-C2 levels and treatment response

First we investigated whether there was a proportional difference in the number of responders and non-responders to oral salmon calcitonin in low and high PRO-C2 subgroups. There were proportionally fewer non-responders in the low PRO-C2 – sCT arm (Chi squared of 6.5, *p* = 0.011) (Table 3).

**Table 3:** Proportion of responders and non-responders in high and low baseline PRO-C2 groups

	Responders mJSN ≤ 0 mm	Intermediate responders 0 < mJSN ≤ 0.35 mm	Non-responders mJSN > 0.35 mm
Low			
Placebo	13 (33)	5 (13)	22 (55)
sCT	14 (45)	8 (26)	9 (29)
High			
Placebo	17 (44)	10 (26)	12 (31)
sCT	14 (38)	9 (24)	14 (38)

Non-responders were defined as those with mJSN > 0.35 mm. Data are shown as number of subjects (%)

mJSN: medial joint space narrowing; sCT: salmon calcitonin

**Fig. 4:**
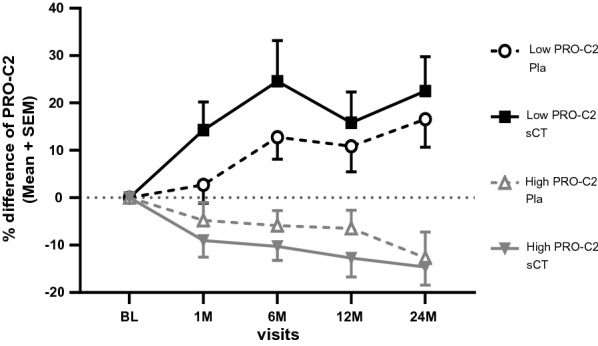
Pharmacodynamic effect of oral salmon calcitonin (sCT) versus placebo (Pla) on serum PRO-C2 levels over time in patients with low and high baseline PRO-C2. Data are shown as the mean (SEM) percentage difference from baseline. Time dependent effects were investigated by ANCOVA adjusting for BMI, sex, age, and baseline medial JSW

Lastly we assessed the pharmacodynamic effect of sCT versus placebo in either low or high PRO-C2 groups. In patients with low baseline PRO-C2, there was an approximate 20% increase in PRO-C2 levels in patients treated with sCT; however, this was not significantly different from the increase (approximately 15%) observed in the placebo (Fig. 4). Both treatment arms in patients with high baseline PRO-C2 showed decreasing levels in PRO-C2 of approximately 15% (Fig. 4). The response to sCT based on PRO-C2 stratification can be found in the supplementary data file (Additional file 5: Fig. S3).

## Discussion

Type II collagen is nearly exclusively localized to cartilage, being the major structural component of this tissue. Recently, immunoassays suitable for measuring serological collagen type IIB N-propeptide fragment (PIIBNP or PRO-C2) were developed and proposed for the estimation of cartilage formation [14]. In the present study, we investigated the clinical utility of PRO-C2. Low baseline PRO-C2 identified a more rapidly progressing subgroup in two independent knee OA clinical cohorts. This suggests that low cartilage formation may be associated with an endotype of higher structural loss. These data agree with results from the FNIH initiative on PIIANP [23], another type II collagen biomarker indicative of collagen formation. In addition, cartilage oligomeric protein (COMP) and urinary CTX-II (uCTX-II) have all shown potential as prognostic biomarkers [23–28]. Interestingly, however, uCTX-II and PIIANP did not provide the same prognostic nor predictive value in the current study (Additional file 4: Fig. S4), and COMP was not investigated. Nevertheless, the fact that PRO-C2 and several other biomarkers, all indicative of joint tissue turnover, are able to predict progression supports the potential to identify one or more clinically relevant endotypes of OA using molecular markers. This is the long-lost promise of precision medicine. Although several initiatives are currently underway to qualify molecular biomarkers for use in OA trials, such as the Applied Public–Private Research enabling Osteoarthritis Clinical Headway (APPROACH) consortium and the FNIH initiative on soluble biomarkers for OA, there is still an urgent need to develop novel and sensitive biomarkers that could be used for categorizing patients, trial enrichment, and personalized healthcare in OA [29, 30].

In the osteoporosis field, it is well known that bone turnover activity is positively associated with the level of response to treatment [31]. In other words, patients with osteoporosis and a high bone turnover respond better to antiresorptive (anticatabolic) [31] and proformation (anabolic) therapies [32]. Additionally, patients with high fibrogenic activity or fibrosis formation (namely rapid progressors) are more likely to respond to an antifibrotic therapy in the liver fibrosis [11]. By analogy to both diseases, a similar approach and paradigm may be applicable to OA and assist in the path towards developing a more endotype specific approach OA therapy, thereby allowing for personalized musculoskeletal health care. Biomarkers related to disease progression and response to a selected intervention would greatly aid the success of drug development in OA. Participants with lower baseline PRO-C2 had a better response to treatment with sCT, as measured by JSN, compared with the placebo group. This suggests that PRO-C2 could serve as a biomarker not only for prediction of OA progression but also for treatment response. We recently demonstrated that FGF-18 induced PRO-C2 in both human and animal chondrocytes [14, 33]. In addition, in the phase II FGF-18 Osteoarthritis Randomized Trial with Administration of Repeated Doses (FORWARD) study, intra-articular FGF-18 demonstrated a dose-dependent increase in the cartilage thickness on magnetic resonance imaging (MRI) [34, 35]. In this trial, synovial fluid PRO-C2 levels increased over time in OA patients treated with FGF18, whereas no change was observed in the placebo arm (unpublished data). Moreover, there were notable differences of cartilage thickness change over 2-years in the total femorotibial joint between OA patients with lower levels of PRO-C2 at baseline and those with high PRO-C2. Compared with the high PRO-C2 subgroup, the low PRO-C2 subgroup demonstrated greater increase in total cartilage thickness and improved outcomes for WOMAC total scores after 2 years of treatment of FGF18 compared with the placebo group [36]. These combined data suggest a causal relationship between PRO-C2 and cartilage formation [37]. It may be possible to extend the treatment response predictability of PRO-C2 to other DMOADs.

**Fig. 5:**
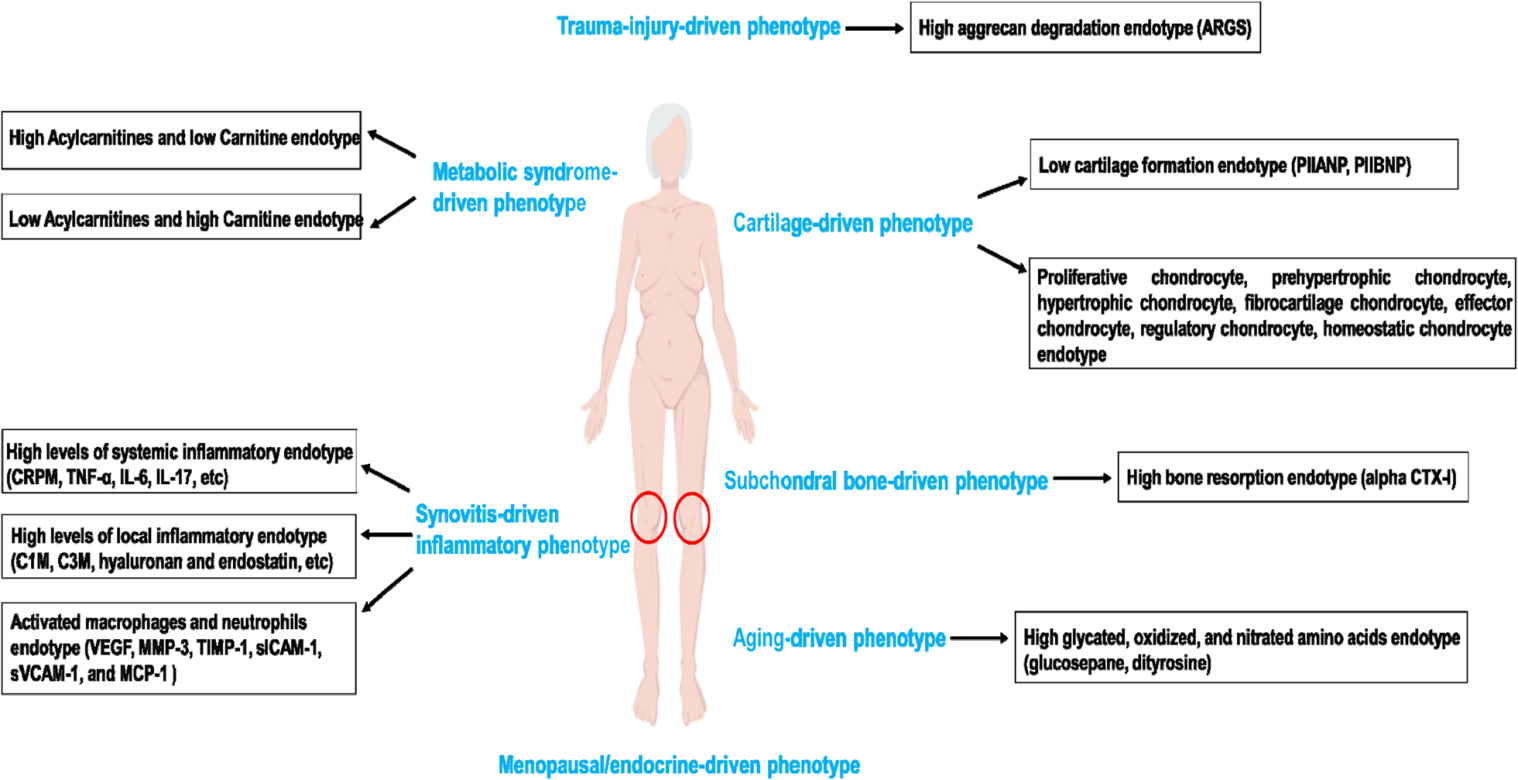
Emerging phenotypes and endotypes of osteoarthritis. ARGS: N-terminal neoepitope of the aggrecanase-mediated aggrecan degradation fragment; C1M: MMP-derived collagen degradation neoepitopes of collagen I; C3M: MMP-derived collagen degradation neoepitopes of collagen III; CRPM: MMP-depended degradation product of C-reactive protein; CTX-I: C-terminal telopeptide of collagen I; IL-6: interleukin-6; IL-17: interleukin-17; MCP-1: monocyte chemoattractant protein 1; MMP-3: matrix metalloproteinase-3; PIIBNP (N-terminal propeptide of procollagens IIB); sICAM-1: soluble intracellular adhesion molecule 1; sVCAM-1: soluble vascular cell adhesion molecule 1; TNF-α: tumor necrosis factor-alpha; TIMP-1: tissue inhibitor of metallopeptidase inhibitor 1; VEGF: vascular endothelial growth factor

OA is a heterogeneous disease, and multiple phenotypes of OA have been identified based on risk factors such as aging, metabolic syndrome, trauma, and endocrine, inflammatory, and subchondral bone-driven progression of OA [38–41]. Some corresponding molecular and/or cellular endotypes have also been reported (Fig. 5), although the terms “endotype” and “phenotype” have been used in confusing ways in different reports. For instance, recent work by Ji et al. investigated the relationships between seven endotypes of OA cartilage chondrocytes and OA severity [42, 43]. They stated that the subgroups of proliferative chondrocytes, pre-hypertrophic chondrocytes, and fibrocartilage chondrocytes were correlated with worse clinical outcomes. The study by Zhang WD et al. identified two metabolic endotypes of knee osteoarthritis that differed in synovial fluid concentrations of acylcarnitine and carnitine [44], which would help to unravel the pathogenesis and develop targeted therapies for OA. In a recent study, glucosepane markedly increased with age and disease progression in both a guinea pig model of knee OA and osteoarthritis in patients [45]. The association of glucosepane with aging-related OA may, for the first time, improve early-stage OA diagnosis and prognosis. Additionally, Huebner et al. observed that a high bone absorption endotype (measured by alpha CTX-I) was associated with OA progression defined by features of JSN and osteophyte in a longitudinal study of patients with symptomatic OA [46]. This indicates that OA treatment should include the targeting of the subchondral bone. In an inflammatory OA phenotype, six synovial fluid biomarkers were recently reported to be specific indicators of an endotype characterized by activated macrophages and neutrophils [47]. In the present study, we discovered a low cartilage formation endotype (measured by the type II collagen formation biomarker, PRO-C2) corresponding to a cartilage-driven phenotype presenting with a higher level of disease progression and superior response to a potential OA treatment. All in all, the progress of understanding the meaning of molecular endotypes and clinical phenotypes could help shed light on the pathophysiological mechanism of OA and aid in patient stratification, better design of clinical trials, and personalized treatments for knee OA patients.

There are several limitations associated with the current study. First, the cut-off values, based on the median concentrations in the two cohorts SMC and NYU, were different (Additional file 2: Fig. S5). This was due to different levels in serum and plasma but also a consequence of distinct clinical characteristics of the two cohorts. The difference seemed to have nothing to do with the fasting versus non-fasting sampling, in that there was no significant difference in PRO-C2 levels between fasting and non-fasting blood from healthy individuals (Additional file 1: Fig. S1). Further validation in larger populations will be needed to determine the specific optimal threshold values for distinguishing OA progression risk. Second, the durations of follow-up for both NYU and SMC cohorts were only 2 years; hence, it remains unknown whether PRO-C2 would predict progression over a longer follow-up period. Third, the number of participants investigated in the longitudinal assessment was relatively small; larger populations (e.g., the entire SMC021-2301 cohort) are required to confirm these findings. Fourth, further studies comparing serum and synovial fluid concentrations of PRO-C2 in well-characterized OA cohorts are warranted to reveal the clinical significance of PRO-C2, considering the fact that synovial fluid is subject to less interference from systemic sources of noise. Fifth, we used only radiographic medial JSW, not MRI, as a surrogate marker for evaluating cartilage degradation of OA progression, as both cohorts were designed one decade ago when MRI features and lateral JSW were not commonly assessed in trials. Lastly, this study examined only knee OA patients, and therefore the results may not be generalizable to patients with other types of OA, e.g., hand and/or hip OA (Additional file 5: Fig. S3).

## Conclusion

In conclusion, these data suggest that endotyping in knee OA patients is feasible. We report that low cartilage formation based on PRO-C2 appears to be a quantifiable OA endotype associated with structural OA progression and response to treatment.

## Supplementary Information

**Additional file 1: Fig. S1.**

**Additional file 2: Fig. S5.**

**Additional file 3: Fig. S2.**

**Additional file 4: Fig. S4.**

**Additional file 5: Fig. S3.**

## Data Availability

The datasets used and/or analysed during the current study are available from the corresponding author on reasonable request.

## Abbreviations

ACR: American College of Rheumatology
ARGS: N-terminal neoepitope of the aggrecanase-mediated aggrecan degradation fragment
BMI: Body mass index
C1M: MMP-derived collagen degradation neoepitopes of collagen I
C3M: MMP-derived collagen degradation neoepitopes of collagen III
COMP: Cartilage oligomeric protein
CRPM: MMP-depended degradation product of C-reactive protein
CTX-II: C-terminal telopeptide of collagen II
CV: Coefficient of variation
DMOAD: Disease-modifying osteoarthritis drug
ECLIA: Electrochemiluminescence immunoassay
ECM: Extracellular matrix
FGF18: Fibroblast growth factor 18
GDF5: Growth differentiation factor 5
IL-6: Interleukin-6
IL-17: Interleukin-17
KLG: Kellgren–Lawrence grades
LLOD: Lower limit of detection
LLOQ: Lower limit of quantification
MCP-1: Monocyte chemoattractant protein 1
mJSW: Medial joint space width
MMP-3: Matrix metalloproteinase-3
MRI: Magnetic resonance imaging
ns: Not significant
OA: Osteoarthritis
PLB: Placebo
PIIANP: Procollagen IIA N-terminal propeptide
PIIBNP: Procollagen IIB N-terminal propeptide
sCT: Salmon calcitonin
SD: Standard deviation
SE: Standard error
SEM: Standard error of mean
sICAM-1: Soluble intracellular adhesion molecule 1
sVCAM-1: Soluble vascular cell adhesion molecule 1
TGF-β1: Transforming growth factor-beta 1
TNF-α: Tumor necrosis factor-alpha
TIMP-1: Tissue inhibitor of metallopeptidase inhibitor 1
VAS: Visual analogue scale
VEGF: Vascular endothelial growth factor
WOMAC: Western Ontario and McMaster Universities Osteoarthritis Index
