# Supplementary material for: A low cartilage formation and repair endotype predicts radiographic progression of symptomatic knee osteoarthritis
Source: J Orthop Traumatol. 2021 Mar 9;22:10. doi: 10.1186/s10195-021-00572-0 (PMC7943687; doi:10.1186/s10195-021-00572-0)
Supplement: Supplementary file 1 — Additional file 1: Fig. S1. [file 10195_2021_572_MOESM1_ESM.docx]

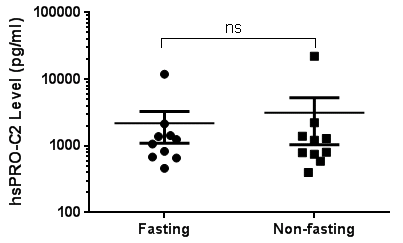


Supplementary S1. hsPRO-C2 difference between fasting and non-fasting serum samples from four healthy men (aged 40 yr) and six healthy women (aged 30.8 yr). Blood samples were drawn from fasting individuals who afterward received a standardized breakfast consisting of 85 g fried sausage, 150 g fried minced beef, 60 g scrambled egg, 50 g fried bacon, and 1 piece of toast. During digestion, only water, tea or coffee was allowed. Non-fasting blood was drawn after 3 hours of digestion. ns, no significance.
