# Supplementary material for: A low cartilage formation and repair endotype predicts radiographic progression of symptomatic knee osteoarthritis
Source: J Orthop Traumatol. 2021 Mar 9;22:10. doi: 10.1186/s10195-021-00572-0 (PMC7943687; doi:10.1186/s10195-021-00572-0)
Supplement: Supplementary file 2 — Additional file 2: Fig. S5. [file 10195_2021_572_MOESM2_ESM.docx]

Supplementary S5. Baseline hsPRO-C2 levels in NYU and SMC cohorts. All values are presented as median and 95% confidence interval (95% CI). The median PRO-C2 values in NYU and SMC cohorts were 1480 pg/mL and 1960 pg/mL respectively.
