# Supplementary material for: A low cartilage formation and repair endotype predicts radiographic progression of symptomatic knee osteoarthritis
Source: J Orthop Traumatol. 2021 Mar 9;22:10. doi: 10.1186/s10195-021-00572-0 (PMC7943687; doi:10.1186/s10195-021-00572-0)
Supplement: Supplementary file 3 — Additional file 3: Fig. S2. [file 10195_2021_572_MOESM3_ESM.docx]

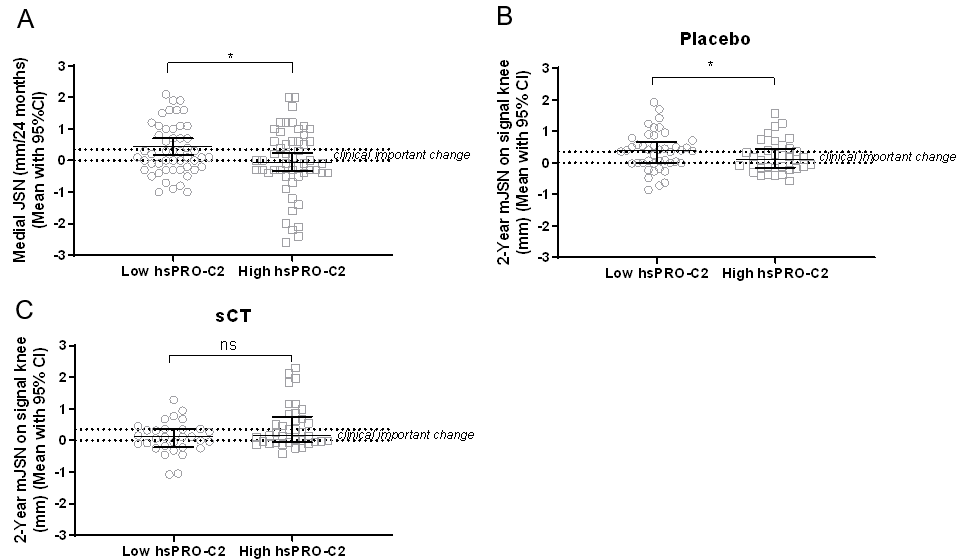


Supplementary S2. Low baseline hsPRO-C2 predicted two-year radiographic progression in the NYU (A) and SMC (B) cohort. All values were compared with Student's two-tailed t-test. Data were adjusted for BMI, sex, age, and baseline medial JSW using two-way analysis of covariates (ANCOVA). Low levels of hsPRO-C2 were those at or below the median in each cohort, whereas the high hsPRO-C2 were above the median. p values represent the significance of the difference between the two groups of baseline levels of molecular markers. Asterisks indicate the following: *p < 0.05. BMI, body mass index; hsPRO-C2, high sensitivity procollagen type IIB N-terminal propeptide; JSN, joint space narrowing; sCT, salmon calcitonin. 0.35 mm JSN is defined as clinical important change.
