# Supplementary material for: A low cartilage formation and repair endotype predicts radiographic progression of symptomatic knee osteoarthritis
Source: J Orthop Traumatol. 2021 Mar 9;22:10. doi: 10.1186/s10195-021-00572-0 (PMC7943687; doi:10.1186/s10195-021-00572-0)
Supplement: Supplementary file 4 — Additional file 4: Fig. S4. [file 10195_2021_572_MOESM4_ESM.docx]

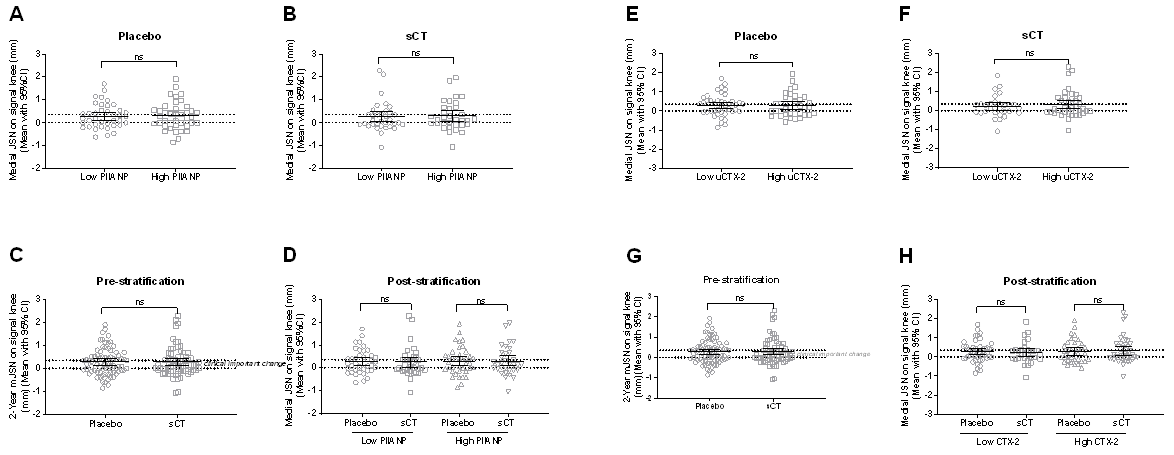


Supplementary S4. Neither baseline serum PIIANP nor baseline urinary CTX-II predicted response to sCT. Two-year radiographic progression of patients with low and high baseline levels (based on the median) of type II collagen synthesis (PIIANP, A-D) and degradation marker (uCTX-II, E-H) at baseline in the SMC cohort. All values are presented as means and 95% confidence interval (95% CI) and were compared with Student’s two-tailed t-test. Data were adjusted for BMI, sex, age, and baseline medial JSW using two-way analysis of covariates (ANCOVA). Low levels of PIIANP and uCTX-II were those at or below the median (781.8 ng/mL, 198 ng/mmoL creatinine, respectively) in the SMC cohort, whereas the high PIIANP and uCTX-II were above the median.
